# Investigating the impact of background noise on collaborative decision-making using an individual-weighted voting model

**DOI:** 10.1186/s41235-026-00710-4

**Published:** 2026-02-27

**Authors:** Ingvi Örnólfsson, Axel Ahrens, Tobias May, Torsten Dau

**Affiliations:** Hearing Systems Section, Department of Health Technology, Technical University of Denmark, 2800 Kgs. Lyngby, Denmark

**Keywords:** Group decision-making, Interactive communication, Task-oriented dialogue, Confidence, Speech-in-noise

## Abstract

The development and evaluation of hearing rehabilitation strategies would greatly benefit from quantification of theoretical constructs related to communication success. Motivated by this, we present a model-based approach to analyze information exchange in a collaborative general knowledge decision-making task. Through a combination of experiments and simulations, we investigate how this model can be used to quantify the exchange of information between interlocutors. Experiments were conducted with ten triads (N = 30) to examine the impact of loud background noise on decision-making in collaborating triads. The group discussions took place in two different levels of background noise, 48dB and 78dB. An existing model of joint decision-making was extended to fit cases where decisions are made individually after engaging in a collaborative discussion. A maximum likelihood estimator for the model was derived and validated in terms of parameter recovery and sensitivity to participant response bias and was used to quantify the relative influence of group members on each other’s post-discussion decisions, formalized as a set of model weights. Four statistics were used to summarize the results: overall weight change, self-weighting, weight equality, and weight similarity. Background noise was found to significantly alter how participants influenced each other’s decisions, but the direction of change remained unclear. These findings demonstrate how group members’ influence on each other’s decisions can be quantified and suggest that loud background noise can have a tangible impact on how group decisions are formed.

**Supplementary Information:**

The online version contains supplementary material available at 10.1186/s41235-026-00710-4.

## Significance statement

Conceptualizing and measuring communication success is crucial for evaluating hearing interventions, as many hearing-impaired individuals struggle in interactive communication settings. However, no established methods exist to directly assess communication success in the context of hearing impairment and intervention. This is arguably due to the elusive nature of this concept; communication success is highly dependent on context and subjectively determined objectives. Nonetheless, one broad conceptual category which encapsulates many everyday communication scenarios is exchange of information. In this study, we adapt an existing model of joint decision-making to operationalize interpersonal exchange of information in groups of arbitrary sizes. We apply this model to an experiment conducted in an environment which is known to make communication difficult—namely, in the presence of loud background noise. We illustrate how the model can reveal changes in the ways in which information is exchanged between interlocutors and confirm that noise does indeed impact information exchange. We illustrate how the model can also be used to make inferences about individuals’ propensity to absorb information from their interlocutors. This methodology offers a new tool for assessing the consequences of communicative inhibitions, such as noise, hearing loss, language proficiency, or speech disabilities. In the context of hearing impairment, this could provide new insights and evaluation criteria which go beyond traditional passive listening tests.

**Supplementary Information:**

The online version contains supplementary material available at 10.1186/s41235-026-00710-4.

## Introduction

An estimated 1.57 billion people worldwide suffer from some degree of hearing loss, with around 400 million experiencing moderate to severe hearing loss (Haile et al., 2021). Existing clinical tools used to measure a patient’s hearing status commonly include measures of pure-tone sensitivity and speech intelligibility in stationary noise, aiming to diagnose hearing loss in isolated, passive listening scenarios. However, the situations in which hearing-impaired listeners struggle the most are very different from these clinical settings. Such situations typically involve the need to communicate interactively with other people, which is known to be particularly difficult for individuals with hearing disabilities (Holman et al., 2019; Kiessling et al., 2003; Nicoras et al., 2023). While a person’s ability to communicate relies strongly on their ability to hear, it is not solely determined by it. Passive listening tests do not reflect the interactive nature of communication and may ultimately be unsuitable for quantifying the capacity for successful communication (Carlile & Keidser, 2020).

The disregard of the interactive elements of communication has its roots in the source-message-channel-receiver model of communication, also known as the linear model of communication (Berlo, 1960). In this model, problems induced by hearing loss are considered to be solely due to a degradation of the signal on the receiver side. Today, it is well known that passively listening to speech is not people’s default or natural mode of communication. Barnlund (1970) provided an early theoretical criticism of the linear model of communication, and numerous experimental studies have since supported his view of communication as fundamentally interactive (Bavelas et al., 2000; Fay et al., 2000; Schober & Clark, 1989). Interaction is a crucial element of successful communication, as elements of the interactive process continuously shape the message itself (Fusaroli & Tylén, 2016; Garrod & Pickering, 2009; Lindblom, 1990). Understanding successful communication thus requires evaluation paradigms that involve interactive dialogue as a central tenet. Despite these established accounts of the interactive nature of dialogue, few advances have been made in terms of quantifying communication success in the context of hearing impairment or other adverse communication situations. A quantitative measure of communication success would be a valuable tool for revealing communication difficulties not adequately captured by non-interactive listening tests typically used to diagnose hearing loss (Carlile & Keidser, 2020).

To measure communication success, it is necessary to define more clearly what the objective of communication is. While such objectives can vary broadly depending on context (O’Connell et al., 1990), one common category is information exchange (Nicoras et al., 2023), which can be quantified and analyzed through collaborative decision-making tasks (Bahrami et al., 2010; Bang et al., 2014; Fay et al., 2000; Keshmirian et al., 2022; Koriat, 2012; Mahmoodi et al., 2015; Meyen et al., 2021). In this framework, participants first make individual decisions about a query, followed by a group discussion round and a second decision round, where participants are asked to reach a consensus decision and/or update their individual decision based on the discussion. Such studies have shown that successful information exchange is not guaranteed, with factors such as group size (Fay et al., 2000; Keshmirian et al., 2022), individual differences in task ability (Bahrami et al., 2010; Bang et al., 2014; Mahmoodi et al., 2015), and linguistic coordination (Dideriksen et al., 2023; Fusaroli & Tylén, 2016) affecting group decision-making. However, this framework has not yet been used to investigate the impact of adverse communication conditions, such as in the presence of hearing impairment or loud background noise. Such conditions can negatively impact communication, so it is plausible that they would also affect information exchange in collaborative decision-making tasks. This approach could potentially be used as a quantitative measure of communication success and/or evaluation of the benefit provided by an intervention, and would ensure a high degree of external validity (Holman et al., 2019; Nicoras et al., 2023).

In this study, we used the group decision-making framework to quantify information exchange in triadic interactions between normal-hearing interlocutors conversing in two different levels of background noise. We used data from a previous study on group decision-making in the presence of background noise (Örnólfsson et al., 2024), which revealed substantial differences in high-level group decision outcomes depending on background noise level. Here, we expand upon this work by analyzing the data through the lens of an explicit statistical model of group decisions, and by proposing four relevant summary statistics which can be tied to different conceptualizations of *communication success*. To achieve this, we adapted an existing model of group decision-making, the so-called confidence weighted majority voting (CWMV) model (Meyen et al., 2021). This model accommodates any group size and is a generalized model that encompasses multiple common strategies employed by groups during decision-making (Grofman et al., 1983; Koriat, 2012). For this study, the CWMV model was slightly modified to allow for an analytical solution to the maximum likelihood estimator and to accommodate individual, non-consensual post-conversation responses. This modification allowed the model to predict one-way information flow, such that one member could influence another to change their decision without the reverse necessarily being true. The rest of this paper is organized as follows. First, we report the methods of our experimental procedure as well as the details of the model and outcome measures used to make inferences about the collaborative decision-making processes observed. Next, we present the results of a series of simulations intended to validate the model, gauge its sensitivity to subjective biases in metacognitive reporting, and aid the interpretation of outcome measures. The results of the experiment are reported in the following section, and the final section discusses the results of both the simulations and the experiment.

## Methods

### Participants & experimental setup

The data analyzed in this study were originally disseminated in another study (Örnólfsson et al., 2024), but we present the main elements of the experimental setup here again for convenience. Ten triads (30 participants) took part in the study. Participants were between 20 and 35 years old and reported having normal hearing. The experiment was conducted in Danish, and all participants were native Danish speakers. The majority of subjects were students at the Technical University of Denmark. When organizing the triads, emphasis was placed on creating mixed-gender groups and ensuring that all three participants were strangers to each other prior to the experiment. However, due to scheduling difficulties, these criteria had to be relaxed. As a result, three groups ended up being same-gender groups, and one group (group C) included a pair of individuals who were acquainted prior to the experiment. The experiment lasted about 2.5 h, and participants were offered hourly monetary compensation for their participation.

During the experiments, participants were seated facing each other in an equilateral triangle, approximately 1.5 m apart. Background noise was played back via an array of eight loudspeakers (Dynaudio BM6P) placed at a distance of 2.4 m from the center. The loudspeakers were driven by a sonible d:24 amplifier, and each one played a Danish monologue (Ahrens & Lund, 2022), resulting in spatially distributed multi-talker noise. The noise was presented at a combined sound pressure level of either 48 dB or 78 dB, referred to as the “quiet” and the “noisy” conditions, respectively. The simultaneous presentation of multiple speech sources rendered them individually unintelligible in both conditions.

### Task

**Fig. 1:**
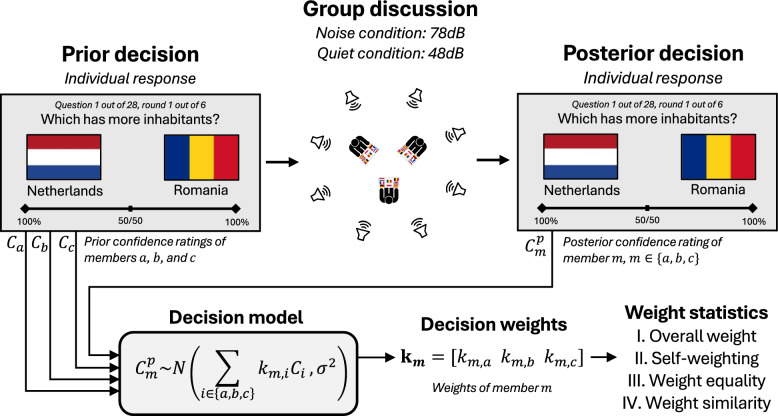
Overview of the experimental procedure and the analysis pipeline. **Prior decision**: Participants initially made individual decisions on a series of binary general knowledge questions, submitting decisions along with a confidence rating using a continuous scale. **Group discussion**: Subsequently, participants engaged in group conversations with two other members, with the aim of improving each other’s answers. The conversation took place either in loud background noise (78 dB spatialized 8-talker babble) or in quiet (48 dB spatialized 8-talker babble). **Posterior decision**: Following the conversation, participants independently and privately repeated the same questions. **Decision model**: Prior and posterior decisions were passed through a formal model of the decision-making process. **Decision weights**: Using maximum likelihood estimation, a set of decision weights was estimated for each participant. **Weight statistics**: Decision weights from the two conditions were analyzed within and across subjects using four summary statistics: overall weight difference, self-weighting, weight equality and weight similarity

Each participant went through three main phases of the experiment, as visualized in Fig. 1. First, participants were asked a series of 28 general knowledge questions on a given topic. Three distinct topics featured in the experiment: Hollywood movies (*Which of these two movies is oldest?*), Copenhagen landmarks (*Which of these two places is closest to the city center?*), and European countries (*Which of these two countries has the larger population?*). For each question, two response alternatives were given, each accompanied by a visual illustration and a label. The 28 questions of each list were formed by combining all unordered pairs of 8 distinct items (i.e., 8 Hollywood movies), resulting in 8*7/2 = 28 pairs of items which were then used as the binary questions for that particular list.

Participants were instructed to select one of the two options and to provide a confidence level, expressed as a percentage between 50 and 100%, with 50% indicating no preference for either option and 100% indicating absolute certainty in the decision. They were instructed to interpret the scale as indicating their estimated probability of having answered the question correctly. After answering the 28 questions, the participants discussed the questions they had just answered in their triad. They were instructed to view the task as a collaborative effort and were told to aim to improve both their own performance and that of their group members, intended to facilitate a free and unstructured exchange of information. Once a 10-min time limit was reached, or the conversation concluded naturally, participants individually answered the same 28 questions again. At the end of the round, participants received feedback in the form of a percent correct score on their pre- and post-discussion responses.

In order to exclude repetition effects, two lists were formed for each topic, one for each noise condition. These lists were kept completely disjoint; no single item (country, movie, or location) would feature in both lists. The process illustrated in Fig. 1 was thus repeated six times, once for each of the three topics and in each of the two noise conditions, each time using a new list. The order of topics and conditions was randomized between groups, with the constraint that the same topic could not appear twice in a row. Prior to the main experiment, the group performed a short trial round on a separate topic not included in the main experiment.

### Group decision model

The group decision model employed was based on the confidence weighted majority voting (CWMV) model (Meyen et al., 2021). The model was originally used in the context of a perceptual task where participants jointly estimated probabilities of biased coin flip sequences. The model predicts a group’s consensus confidence rating, ${c}_{g}$, in a binary decision scenario where each individual member’s prior confidence is known. Given prior confidence ratings, ${c}_{i}$, from $M$ individuals, ${C}_{g}$ is predicted to be:

1 $$\begin{array}{c}{C}_{g}\sim N\left(k{\sum }_{i=1}^{M}{C}_{i},\sigma \right)\end{array}$$

The confidence ratings ${C}_{g}$ and ${C}_{i}$ are measured in log-odds units, such that $C={\mathrm{ln}}\left(\frac{c}{1-c}\right)$, where $c$ is a confidence rating on a bounded scale from 0 to 1. Here, $c=0 \Rightarrow C =-\infty$ indicates maximal confidence in one option, $c=1 \Rightarrow C = \infty$ indicates maximal confidence in the other option, and $c=0.5 \Rightarrow C = 0$ indicates no preference for either option. The parameters $k$ and $\sigma$ are free parameters that control the shape of the probability distribution of posterior confidences given a set of $M$ prior confidence ratings. Note that the definition given here differs slightly from the one originally proposed, as the errors are assumed to be normally distributed around ${C}_{g}$, and not ${c}_{g}$. Furthermore, an expansion coefficient for the confidence ratings has been dropped for simplicity.

One necessary adaptation of the CWMV model is that it predicts a single consensus decision, which is not the case in the present study. To allow for individual posterior decisions, ${C}_{g}$ was replaced with ${C}_{j}^{p}$, representing the posterior (indicated by the superscript $p$) confidence of member $j$. Furthermore, different weights were included for each pair of group members by adding indices $i$ and $j$ to the free parameter $k$. This gives the decision model used in this study:

2 $$\begin{array}{c}{C}_{j}^{p}\sim N\left({\sum }_{i\in a,b,c}{k}_{j,i}{C}_{i},\upsigma \right)\end{array}$$

To emphasize that we explicitly consider triadic groups in this study, $i$ is presented as a categorical variable representing the three group members, $a$, $b$, and $c$, but the model is still in principle applicable to any group size. In this model, the free parameter ${k}_{j,i}$ acts as a weighting factor on the initial confidence ratings ${C}_{i}$. The weighting factor ${k}_{j,i}$ controls how much participant $j$ is influenced by group member $i$’s prior confidence rating when making their own posterior decision. Using individual weights for each group member allows the model to account for the fact that individual members might receive more or less information from each other due to factors like hearing status, susceptibility to noise, personality factors, etc. The model weights ${\boldsymbol{k}}$ were estimated using an analytically derived maximum likelihood estimate. Given $N$ trials of prior and posterior confidences from three group members, a maximum likelihood estimate for the weight vector ${{\boldsymbol{k}}}_{j}$ can be derived from the following system of equations (see Supplementary Materials for derivation details):

3 $$\begin{aligned} \sum\limits_{{n = 1}}^{N} {\left( {\left[ \left \{ {\begin{array}{*{20}c} {C_{{a,n}} } \\ {C_{{b,n}} } \\ {C_{{c,n}} } \\ \end{array} } \right. \right] \cdot \left[ \left \{ {\begin{array}{*{20}c} {C_{{a,n}} } \\ {C_{{b,n}} } \\ {C_{{c,n}} } \\ \end{array} } \right. \right]^{T} \cdot \left[ \left \{ {\begin{array}{*{20}c} {k_{{j,a}} } \\ {k_{{j,b}} } \\ {k_{{j,c}} } \\ \end{array} } \right. \right]} \right)} = \sum\limits_{{n = 1}}^{N} {\left( {\begin{array}{*{20}c} {C_{{a,n}} C_{{j,n}}^{p} } \\ {C_{{b,n}} C_{{j,n}}^{p} } \\ {C_{{c,n}} C_{{j,n}}^{p} } \\ \end{array} } \right)} \\ \end{aligned}$$

For clarity, the summation operators are taken to act on each row separately. ${C}_{i,n}$ denotes the prior confidence of member $i$ in trial $n$, and ${C}_{j,n}^{p}$ is the posterior confidence of member $j$ in trial $n$. Given observations of $C$ and ${C}_{j}^{p}$, this system of equations can be solved for $${{\boldsymbol{k}}}_{j}=\boldsymbol{ }{\left[\begin{array}{ccc}{k}_{j,a}& {k}_{j,b}& {k}_{j,c}\end{array}\right]}^{T}$$, the weight vector of a given member $j$. When estimating weights from observed data, the weights are assumed to be invariant across multiple decisions; $N$ distinct decisions are used to estimate each group member’s weight ${{\boldsymbol{k}}}_{j}$. Separate weights were estimated from observations from each of the two noise conditions. When estimating weights using data from the experiment, trials with a confidence rating of 100% were first truncated to 99% to prevent infinite values when converting the confidence ratings to the log-odds domain. This effectively limited the magnitude of confidence scale in the log-odds domain to $\pm \frac{0.99}{1-0.99}\approx \pm 4.60$.

### Weight distances

To make quantitative claims about the effect of an intervention on the information exchange weights, a meaningful measure of distances between weights is required. We used the cosine distance to quantify the distance $D$ between weight vectors, i.e., $D\left({\boldsymbol{x}},{\boldsymbol{y}}\right)=\frac{{\boldsymbol{x}}\cdot {\boldsymbol{y}}}{\left|{\boldsymbol{x}}\right|\left|{\boldsymbol{y}}\right|}$. Results are reported in radians, corresponding to the angle between two weight vectors.

**Fig. 2:**
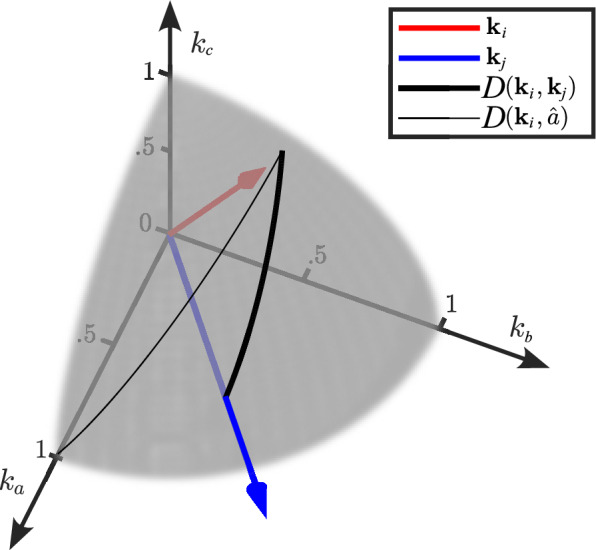
Illustration of angular distances between weights and axes. Each of the three axes corresponds to the estimated weight toward group members a, b, and c, respectively. Two hypothetical weights are shown as the red and blue arrows. The gray hemisphere represents the part of the unit sphere where all weights are nonnegative; a weight vector in this area represents a set of posterior decisions that are a compromise between the prior decisions. The black lines represent the angular distance between two weights (bold) and between a weight and an axis (slim)

Two uses of the cosine distance are illustrated in Fig. 2. Two hypothetical weight vectors, ${{\boldsymbol{k}}}_{i}$ and ${{\boldsymbol{k}}}_{j}$, belonging to members $i$ and $j$, are shown in blue and red, respectively. The bold line shows the cosine distance between the two weights, i.e., $D\left({{\boldsymbol{k}}}_{i},{{\boldsymbol{k}}}_{j}\right)$. The thin line shows the cosine distance between a weight vector and the axis “belonging” to member $a$. The axes can each be thought of as "belonging" to a specific group member, as each dimension represents the weight toward a certain member. Assuming nonnegative weights, the maximum possible cosine distance between weights is $\frac{\uppi }{2}$ rad, which occurs only if at least one of the weights is parallel to an axis.

### Weight distance summary statistics

The possible directions of weight vectors spanned by nonnegative weights are illustrated as the gray hemisphere in Fig. 2. The weights provide a quantified representation of how information is exchanged between individuals in a particular group. To facilitate comparison across multiple groups, four summary statistics were defined based on the information exchange weights and the angular distances between them. These summary statistics—overall weight change, self-weighting, weight equality, and weight similarity, introduced separately in the following—are each associated with a different view on what constitutes successful information exchange, providing complementary perspectives on how to interpret the weights estimated using the decision model.

The first summary statistic, overall weight change, was quantified as $D\left({{\boldsymbol{k}}}_{N},{{\boldsymbol{k}}}_{Q}\right)$, where ${{\boldsymbol{k}}}_{N}$ and ${{\boldsymbol{k}}}_{Q}$ denote the noise and quiet condition weights, respectively, for any given participant. This statistic was motivated by the idea that the quiet condition may be thought of as representing an “ideal” communication scenario, where no inhibitive effects on communication are present. In this view, any substantial change in weights away from the quiet condition represents a detriment to the information exchange process.

The second summary statistic, self-weighting, was defined using the relative weight toward oneself, i.e., $D\left({{\boldsymbol{k}}}_{a},\widehat{{\boldsymbol{a}}}\right)$ for the self-weighting of some group member $a$. A low value of $D\left({{\boldsymbol{k}}}_{a},\widehat{{\boldsymbol{a}}}\right)$ indicates a high degree of self-weighting. Self-weighting quantifies how much new information the participant receives during the experiment. Reliability of external sources is crucial to decision-making task where multiple sources have to be integrated, such as in the present experiment (Nurse et al., 2011; Park et al., 2023). As the noise level increases from quiet to loud noise, information from other group members gradually becomes less reliable as the noise increases, which should lead to an increased self-weighting.

The third summary statistic used was weight equality. Defining the uniform weight $\widehat{{\boldsymbol{u}}}=\left[1\hspace{1em}1\hspace{1em}1\right]$, the distance $D\left({\boldsymbol{k}},\widehat{{\boldsymbol{u}}}\right)$ quantifies the distance to this uniform weight, with low $D\left({\boldsymbol{k}},\widehat{{\boldsymbol{u}}}\right)$ indicating high weight equality. This statistic is motivated by mathematical considerations about the optimal weights that members of a group can use to combine information in decision-making tasks, which has been shown to be proportional to the log-odds of the probability of being correct (Grofman et al., 1983; Marshall et al., 2017). If the confidence ratings $c$ provided by participants reflect their probability of being correct on a given trial, the ideal value of the weight vector $k$ in the present model would thus be a uniform weight. If noise impacts information exchange negatively, weight equality should thus be expected to be higher in quiet conditions.

The fourth and final summary statistic used was weight similarity. Weight similarity was quantified by the distance between the weights of each pair of individuals, i.e., $D\left({{\boldsymbol{k}}}_{a},{{\boldsymbol{k}}}_{b}\right)$, $D\left({{\boldsymbol{k}}}_{a},{{\boldsymbol{k}}}_{c}\right)$ and $D\left({{\boldsymbol{k}}}_{b},{{\boldsymbol{k}}}_{c}\right)$, with low values indicating more similar weights. Similar weights could indicate agreement on the metacognitive sensitivity of each member, which is related to successful decision-making in groups (Bahrami et al., 2010; Bang et al., 2017). This can be considered a more general form of the weight equality statistic, where the criterion that weights must converge toward $\widehat{{\boldsymbol{u}}}=\left[1\hspace{1em}1\hspace{1em}1\right]$ is relaxed. Instead, optimal information exchange is achieved when weights converge at *any* point in $k$-space.

### Statistical analysis

The four weight change statistics were compared between the two conditions using permutation tests. For individual-level analysis, permuted samples were created by randomly shuffling the noise and quiet labels 10,000 times for each participant’s confidence ratings. New weights were estimated in each permuted sample, and permuted summary statistics were calculated using these weights. All tests were two-tailed, except for the test of the overall weight change statistic, which was one-tailed, since the statistic in question was nonnegative by definition. For group- and population-level analyses, outcomes were summarized as the median value across participants. Population-level outcomes were additionally tested with intermediate pooling at the group level to account for potential dependence within groups. All error bars reported span the 2.5th through 97.5th percentiles, computed by bootstrapping.

## Model validation

### Parameter recovery

**Fig. 3:**
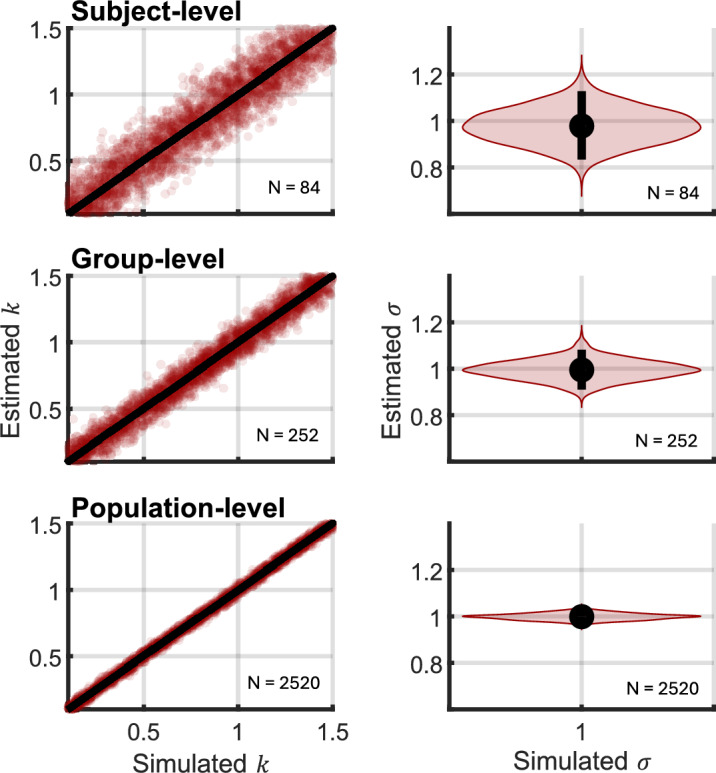
Parameter recovery analysis. Data were simulated from the proposed model and parameters estimated using the analytical maximum likelihood function. Each component of ${\boldsymbol{k}}$ is plotted independently on the same axis. The simulated value of the variance parameter $\upsigma =1$ corresponds roughly to what was estimated from the data in the main analysis (see Results)

To validate the estimates of weight vectors, a parameter recovery analysis was conducted. Simulated data were generated with ${\boldsymbol{C}}$ randomized to mimic the population-level distribution observed in the empirical data, and each with each component of the vector ${\boldsymbol{k}}$ uniformly and independently distributed between 0.1 and 1.5; 1000 random draws of these variables were simulated. $\sigma$ was fixed to 1, corresponding closely to what was later estimated using the empirical data. The number of trials used for the simulations corresponded to the number of trials used in the experiment at the subject, group, and population levels, respectively. The results, shown in Fig. 3, verified that model parameters were recoverable.

### Validation of distance metrics

**Fig. 4:**
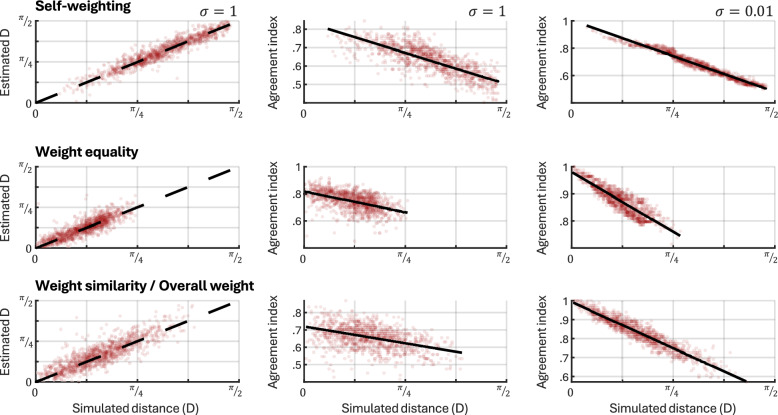
Validation of distance measures. The first column shows recovery of true distance measures using simulated values of ${\boldsymbol{k}}$. Estimated values correspond well with the underlying simulated values. The second and third columns show how each distance measure compares to a simple index of agreement with a relevant baseline (see full text for details). Higher distances correlate with lower agreement indices, verifying that the four distance metrics capture the intended effects

To validate the weight distance metrics, the parameter recovery analysis was extended to include these metrics. The first column of Fig. 4 shows each of these distance metrics calculated using the simulated and estimated ${\boldsymbol{k}}$, respectively, and indicate good recovery. Weight similarity and overall weight difference were both computed in the same manner—by using pairs of random ${\boldsymbol{k}}$—so these measures were treated as a single category for this analysis. To ensure that the distance metrics captured the effects that they were intended to, they were also validated against simple *agreement indices*. Each agreement index is derived only based on which of the two options was picked on any trial, i.e., the signs of $C$ and ${C}^{p}$. For self-weighting, the agreement index is computed as the fraction of trials where the $\mathrm{sign}({C}^{p})=\mathrm{sign}({C}_{a})$, where $a$ is the member whose self-weighting is being compared. For weight equality, the agreement index is the fraction of trials where $\mathrm{sign}({C}^{p})=\mathrm{sign}(\Sigma {\boldsymbol{C}})$, i.e., how often the decision agrees with what would be obtained from equal weighting. For weight similarity/overall weight difference, the agreement index is the fraction of trials where the sign of $\mathrm{sign}\left({C}^{p,1}\right)={\mathrm{sign}(C}^{p,2})$, where the added superscript denotes the two separate random draws of ${\boldsymbol{k}}$. The results are shown in the second and third columns of Fig. 4, using $\sigma =1$ (comparable to the variance later estimated in the dataset) and $\sigma =0.01$, respectively. Each distance metric is negatively correlated with its agreement index, verifying that increased distances indicate decisions that are less aligned with that particular baseline.

### Effect of biased confidence reporting

**Fig. 5:**
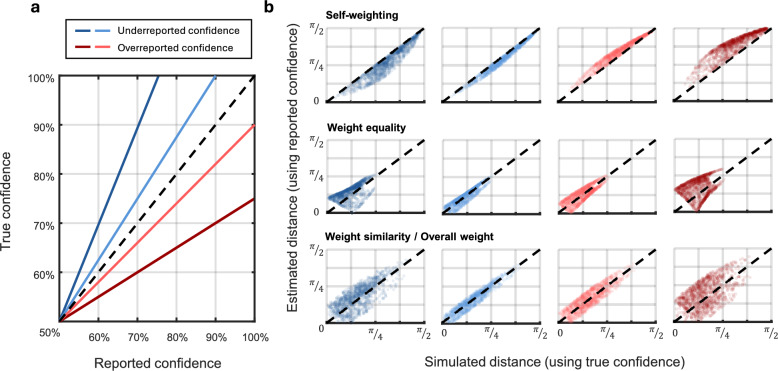
Simulating the effects of over- and underreported confidence. **a** The transformations used to simulate reporting bias. Blue graphs represent underreporting of the true (latent) confidence, red graphs show overreporting. **b** The effect of reporting biases on the estimated distance measures. True confidence values are transformed using the mappings shown in **a** before parameter estimation, leading to biased results for self-weighting and increased variance for all measures. The impact on inference quality increases with the severity of the reporting bias (light vs. dark colors)

A reasonable concern when modeling combined confidence ratings by summation, as the present model does, is how response biases affect model estimates. It is assumed that a latent sense of confidence maps monotonically to the confidence report ${C}_{i}$, but certain differences in this mapping are to be expected across individuals. As true confidence is an inaccessible latent state, the model relies on confidence reports as a proxy, but if this proxy is biased differently for different individuals, it may interfere with model inference. To investigate this phenomenon, we conducted a series of simulations. Once again, simulated data were generated with ${\boldsymbol{C}}$ randomized to mimic the population-level distribution observed in the empirical data and each component of ${\boldsymbol{k}}$ uniformly distributed between 0.1 and 1.5. $\sigma$ was fixed to 0.01 to isolate variance related to response bias differences. Response bias differences were emulated by biasing one member’s reported confidence using a linear mapping. Four different settings were used (Fig. 5a); two degrees of overreported confidence (blue), and two degrees of underreported confidence (red), with each degree corresponding roughly to common and extreme biases observed in the present study (see Supplementary Materials). We computed weight distance metrics for each setting, shown in Fig. 5b. The effect of response bias on self-weighting is shown in the top row. Note that the member whose confidence reports were biased is not the same member as the one whose self-weighting is reported; the effect on self-weighting is thus under the assumption that someone else’s responses are biased. This simulation shows that another’s response bias leads to biased estimates of self-weighting; when some member is underreporting confidence, other members’ self-weighting is underestimated. For the remaining outcome measures, the interaction was more complex. Weight equality seemed to bifurcate into two separate cases, with either over- or underestimation; informal exploration of the model’s behavior suggested this to be related to the magnitude of ${\boldsymbol{k}}$. For weight similarity/overall weight difference, the result of response bias was an increase in the variance of the estimate. Overall, these simulations show that response bias can leak into estimates of the weight distance measures, but that minor response biases should not be problematic for inference.

## Results

### Estimated information exchange weights

**Fig. 6:**
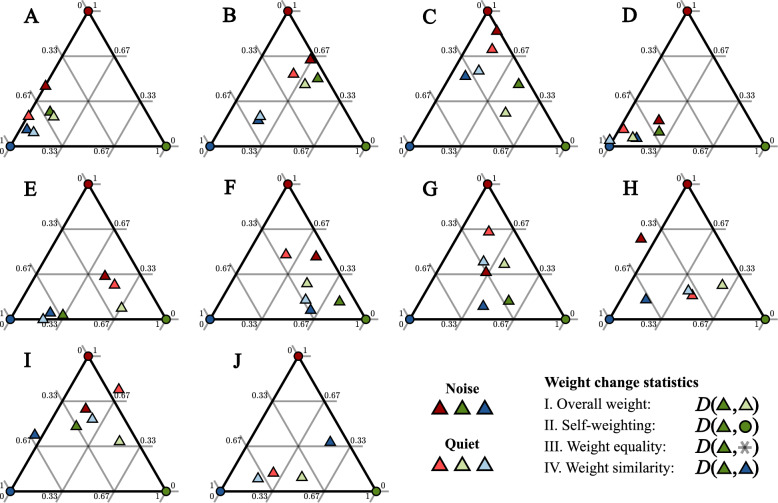
Relative size of weights ${\mathbf{k}}_{\mathbf{i}\mathbf{m}}$ of member $\mathrm{i}$ (indicated by color) toward each group member. Each diagram shows a separate group. The center of each diagram indicates equal weight toward all members, whereas the corners indicate high relative weight toward one particular member and low relative weight toward the other two members. The colors of the corners indicate the "owner" of that corner; for example, a weight close to the green corner indicates a high relative weight toward the green member. The four weight change statistics are illustrated in the bottom right corner

The weight vectors estimated by the model are reported in the Supplementary Materials and are summarized in Fig. 6. Each of the ten subplots represents a single group, with each color (red, blue and green) representing a given member within that group. Each corner of the triangular grid represents a point where the weight vector is nonzero only for the member indicated by the color of the circle. The center of the diagram represents the uniform weight, where all three components of the weight vector have the same magnitude. Each weight is represented by a colored, triangular marker, with dark-shaded weights showing the noise condition, and light-shaded weights the quiet condition. This two-dimensional representation of the weights is achieved by normalizing each weight to sum to one. Compared to the visualization shown in Fig. 2, the diagrams in Fig. 6 correspond to a two-dimensional planar slice through the points ${k}_{a}=1$, ${k}_{b}=1$, and ${k}_{c}=1$, i.e., the plane where ${k}_{a}+{k}_{b}+{k}_{c}=1$. The straight-line distance between two weight vectors in Fig. 3 is thus a reasonable indicator of the three-dimensional cosine distances between those weights.

Some weights are shown outside the bounds of the ternary plots, which indicates negative weights. In theory, this would mean that the member in question is deliberately going against the decisions of the member toward whom they have a negative weight. All the negative weights visible in Fig. 6 are close to zero (i.e., close to an edge of the diagram), indicating that these are most likely statistical anomalies. However, three weights have large enough negative components to not be visible (H-green-noise, J-red-noise, and J-green-noise). These groups were considered potential outlier groups, and any significant population-level effects were checked both with and without these groups included.

### Weight change statistics

**Fig. 7:**
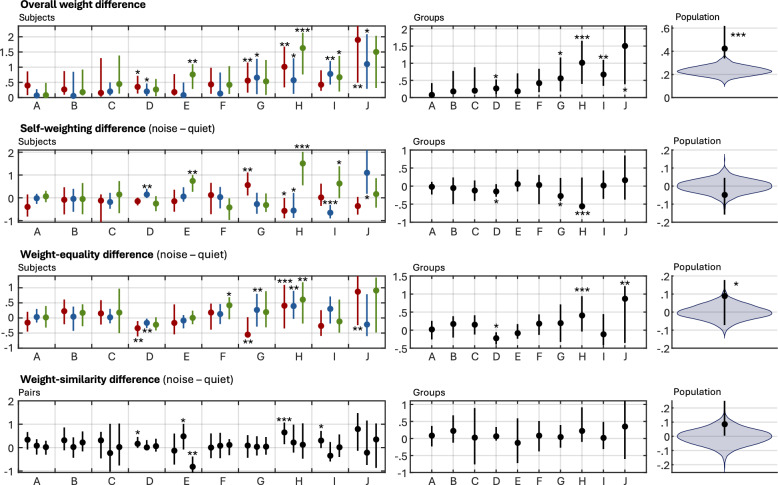
The four summary statistics of the decision weights (organized vertically) for different stratification levels (organized horizontally). For the overall weight statistic, the reported value indicates the absolute distance between weights in the two conditions. For the remaining three statistics, positive values indicate larger distances in noise. For the population-level measures, the violin plot shows the distribution of the statistic under a null hypothesis where condition labels were randomly permuted

The four weight change summary statistics were derived from the estimated weights of each participant and are shown in Fig. 7. For the self-weighting, weight equality, and weight similarity statistics, positive values indicate larger distances in noise, and thus larger *similarity* to their respective baselines in quiet. Stratification by group and population levels are shown in the second and third columns, respectively.

The overall weight change between conditions was found to be significant for 12 participants and for four groups. The population-level median change was $0.42$ rad (bootstrapped 95% CI using the percentile method: [0.35 0.60]) and was strongly significant ($p<0.001$ for $N = \mathrm{10,000}$ permutations). This finding was robust to the omission of the potential outlier groups H and J ($p = 0.006$ when these groups were omitted), and to intermediate pooling at the group level ($p=0.016$).

Changes in self-weighting were significant for ten participants. However, the direction of change varied, with four participants showing higher self-weighting in noise, while six had higher self-weighting in quiet. Three groups had significantly higher self-weighting in noise. The population-level median change was $-0.049$ rad (95% CI [− 0.17 0.056]), but this difference was not significant ($p = 0.24$*,* $N = \mathrm{10,000}$).

Weight equality changed significantly between conditions for nine participants, with six showing increased equality in quiet and three showing increased equality in noise. Three groups showed significantly increased equality in quiet. The population-level median change was $0.089$ rad (95% CI: [-0.051 0.16]), indicating a general tendency for higher equality in quiet ($p = .012$*,* $N = \mathrm{10,000}$). This effect was robust to intermediate pooling at the group level ($p<0.001$), but not to the omission of the potential outlier groups H and J ($p = .417$*,* $N = \mathrm{10,000}$).

Weight similarity changed significantly between conditions for five participants, four showing increased similarity in quiet and one showing increased similarity in noise. No significant differences were found for any groups, and there was no significant population-level difference ($p = .082$*,* $N = \mathrm{10,000}$).

## Discussion

### Main findings

While several previous studies have shown how noise impacts the conversational behavior of interlocutors (Hadley et al., 2019; Miles et al., 2023; Örnólfsson et al., 2023; Sørensen & Fereczkowski, 2019), this study introduces a new way of framing communication success by analyzing group decision-making, specifically through the decision weights individuals use to integrate information from each other into their post-conversation decisions. Although the estimated weights varied widely across participants and groups, some general observations were still possible. First, the model almost exclusively estimated positive weights in both conditions. This suggests that the conversations led to collaborative information exchange, as participants’ post-discussion decisions converged toward a compromise between their prior decisions. Second, one of the proposed summary statistics, the overall weight change between conditions, was significantly larger than what would be expected by chance at the population level. The observed median difference between conditions was 0.42 radians, corresponding to 27% of the maximum distance ($\frac{\pi }{2}$) within the positive octant of $k$-space. However, some nonzero variation is to be expected by chance, as this statistic is strictly nonnegative (c.f. the population-level permuted null distribution shown in Fig. 7, top right). In the simulation presented in Fig. 4, 0.42 rad corresponds to, on average, 5.3% decrease in the disagreement index. This gives an indication of the practical magnitude of the effects observed, although it should be emphasized that the agreement index does not take into account the confidence ratings of individuals, and that any comparison between these metrics is contingent on the assumptions about the underlying data generating process, i.e., the distributions of ${\boldsymbol{C}}$ and ${\boldsymbol{k}}$. Regardless, the overall weight difference was larger than what would be expected by chance, indicating that noise generally impacted the relative weighting scheme applied by participants. The effect was very clear for some groups and participants (e.g., groups H, I and J), while others did not seem to change their weights in response to the noise (e.g., groups A, B, and C). This suggests that even young normal-hearing subjects exhibit individual differences in noise susceptibility during collaborative information exchange.

Of the three remaining summary statistics, only weight equality was significantly impacted by the noise condition. However, this finding was not robust to the omission of the two groups with high negative weights (H and J), indicating no strong evidence for a general effect of noise on weight equality. It is noteworthy that all three statistics nonetheless changed in the direction predicted by the assumptions motivating their inclusion in this study: self-weighting was higher in noise, and weight equality and similarity were higher in quiet. This suggests there may be some merit to the ideas motivating these statistics which could be investigated further.

The methodology and experiment presented here demonstrate that information exchange can be quantified using a group decision-making task and a formal group decision model. However, it remains unclear what direction of changes can be expected when communication is made more difficult. Different groups may follow different weight change patterns in response to communicative interventions or inhibitions. It is also possible that different types of interventions could lead to different changes in decision-making behavior. Care should be taken to define a priori what changes are expected based on the intervention in question.

### Model limitations

The extended CWMV model presented here provides a starting point for model-based quantification of information exchange between individuals in a conversation, but there are some notable limitations in its current form. First, the model assumes that the combination of information from the three group members is unaffected by the agreement status of the group. A trial where the group starts by agreeing is assumed to use the same weights as a trial where there is initial disagreement, but it is likely that a majority vote has some benefit beyond the mere summing of confidence ratings (Schulz-Hardt & Mojzisch, 2012). Another limitation of the model is that it does not account for individual biases in using the confidence scale. Our simulations showed that differences in individual reporting biases may interfere with inferences about weight statistics, but the model cannot account for these. Since latent confidence cannot be measured directly, it is difficult to gauge exactly how severe this bias is. One comparison point could be metacognitive calibration of individuals, i.e., assuming that latent confidence is correlated with the probability of being correct. If this is the case, our sample is largely comparable to the mild cases shown in light blue and light red in Fig. 5, where the additional bias and variance of the distance measures is small (see Supplementary Materials for test participants’ metacognitive calibration curves). Nonetheless, future work could attempt to address the issue of interpersonal calibration directly, possibly through a combination of a separate calibration task and an appropriate model that can capture idiosyncratic differences in calibration (Zhang & Maloney, 2012).

### Experimental limitations

There are also experimental aspects of the current task implementation that may contribute to unexplained variance in the weight estimates. One example is the memory capacity required to solve the task. Since the 28 questions in each list are discussed simultaneously, participants need to keep all these questions and the exchanged information in mind while making their post-discussion decisions. There is likely some information loss between the conversation and the posterior decision, especially related to new information obtained from other members. This could result in participants regressing back toward their own prior beliefs if they forget what they learned in the discussion round. Discussing a long list of related items also has another drawback: the transitivity of the relationship between items. For example, in a list containing items A, B, and C, if one group member knows that A > B and another knows that B > C, they will be able to infer A > C deductively by combining their individually held information, even if both were previously agnostic about the relationship between A and C. This would result in high posterior confidence even though the prior confidences were zero, which the current model cannot account for. The use of general knowledge questions to elicit confidence ratings also risks creating a situation where one or more participants have little or nothing to contribute. Using perceptual stimuli to precondition participants’ confidence ratings may be a more robust approach (Bang et al., 2017; Dideriksen et al., 2023). Nevertheless, there are certain benefits of using general knowledge questions, as they require no preconditioning and, anecdotally, we note that they seemed to produce lively and naturalistic discussions in the groups observed in this study.

## Conclusion

Using a model of collaborative decision-making to analyze an existing dataset of collaborative decision-making in triads, we showed that group members change the weight with which they influence each other’s decisions when they are subjected to loud background noise. We did not identify any common patterns specifying the direction of this change but rather observed substantial interpersonal differences in the direction of change. The methodology presented in this study provides a foundation for developing a framework to assess information exchange, which is an important factor contributing to communication success. Quantifying an individual’s capacity to participate in group decision-making may offer a meaningful way to evaluate the quality of a communication scenario. In a broader context, the results also reveal nuances in how groups of individuals influence each other in different levels of background noise, which could have implications for how we think about verbally mediated collaboration in, e.g., noisy workplace environments.

## Supplementary Information

Supplementary Material 1.

## Data Availability

The raw confidence and decision data gathered from participants in this study is publicly available from DTU Data at 10.11583/DTU.25163816. Tabulated estimates of the weights and their summary statistics for individual participants are available at 10.11583/DTU.25943116.
