# Supplementary material for: Investigating the impact of background noise on collaborative decision-making using an individual-weighted voting model
Source: Cogn Res Princ Implic. 2026 Feb 27;11:17. doi: 10.1186/s41235-026-00710-4 (PMC12946328; doi:10.1186/s41235-026-00710-4)
Supplement: Supplementary file 1 — Supplementary Material 1. [file 41235_2026_710_MOESM1_ESM.docx]

## Derivation of maximum likelihood estimator

Given $N$ observations of $C^{p}$ and $C$, the log-likelihood function for the normal distribution of posterior confidences of group member $i$, as defined in equation $(2)$ of the main article, is

$$\begin{aligned} l\left( \mathbf{k}_{\mathbf{i}},\sigma_{i}^{2} | C_{i,n}^{p} \right)=-\frac{N}{2}\ln2\pi\sigma_{i}^{2}-\frac{1}{2{\sigma_{i}}^{2}}\sum_{n=1}^{N} \left( C_{i,n}^{p}-\mu\right)^{2}\#\left( A.1 \right) \end{aligned}$$

where

$$\begin{aligned} \mu=\sum_{m\in a,b,c} k_{i,m}C_{m,n}\#\left( A.2 \right) \end{aligned}$$

Limiting the derivation to an estimator for the weights of a particular member, the index $i$ can be dropped for clarity:

$$\begin{aligned} l\left( \mathbf{k},\sigma^{2} | C_{n}^{p} \right)=-\frac{N}{2}\ln2\pi\sigma^{2}-\frac{1}{2\sigma^{2}}\sum_{n=1}^{N} \left( C_{n}^{p}-\sum_{m=1}^{M} k_{m}C_{nm} \right)^{2}\#\left( A.3 \right) \end{aligned}$$

Here, $k_{m}$ thus denotes the weight towards member $m$ by the member whose posterior confidence ratings are $C_{n}^{p}$. To solve for $\mathbf{k}$, we first differentiate with respect to the weight $k_{a}$ towards a particular member $a$, and equate the result to zero:

$$\begin{aligned} 0=\frac{\partial l\left( \mathbf{k},\sigma^{2} | C_{n}^{p} \right)}{\partial k_{a}}\#\left( A.4 \right) \end{aligned}$$

$$\begin{aligned} 0=\frac{1}{\sigma^{2}}\sum_{n=1}^{N} \left( C_{a,n}\left( C_{n}^{p}-\sum_{m\in a,b,c} k_{m}C_{m,n} \right) \right)\#\left( A.5 \right) \end{aligned}$$

$$\begin{aligned} 0=\sum_{n=1}^{N} C_{a,n}C_{n}^{p}-\sum_{n=1}^{N} C_{a,n}\sum_{m\in a,b,c} k_{m}C_{m,n}\#\left( A.6 \right) \end{aligned}$$

Expanding the sum over $M$ gives:

$$\begin{aligned} \sum_{n=1}^{N} C_{a,n}C_{n}^{p}=k_{a}\sum_{n=1}^{N} C_{a,n}C_{a,n}+k_{b}\sum_{n=1}^{N} C_{a,n}C_{b,n}+k_{c}\sum_{n=1}^{N} C_{a,n}C_{c,n}\#\left( A.7 \right) \end{aligned}$$

Taking the derivative with respect to the weights towards members $b$ and $c$ results in two additional equations, which are identical to equation $(A.7)$, except that index $a$ becomes $b$ and $c$, respectively. This gives the following system of equations:

$$\begin{aligned} \sum_{n=1}^{N} \left( \left[ \begin{matrix} C_{a,n} \\ C_{b,n} \\ C_{c,n} \end{matrix} \right]\cdot\left[ \begin{matrix} C_{a,n} \\ C_{b,n} \\ C_{c,n} \end{matrix} \right]^{T}\cdot\left[ \begin{matrix} k_{a} \\ k_{b} \\ k_{c} \end{matrix} \right] \right)=\sum_{n=1}^{N} \left( \begin{matrix} C_{a,n}C_{n}^{p} \\ C_{b,n}C_{n}^{p} \\ C_{c,n}C_{n}^{p} \end{matrix} \right)\#\left( A.8 \right) \end{aligned}$$

where the summation operator is taken to act on each row separately. Equation $(A.8)$ is identical to equation $(3)$ in the main article, and can be solved for $\mathbf{k}$, yielding the maximum likelihood estimate of the weights.

## Confidence ratings

The confidence ratings submitted by each participant are shown in Figure 1. Groups are separated by the dashed vertical lines, and the color indicates participant identity (blue for the first member, red for the second, and green for the third). The violin plots show the distribution of log-odds transformed confidences, $C$, across all conditions and lists, separated into prior (upper panel) and posterior responses (middle panel). The horizontal line indicates the median of each participant’s confidence ratings. The lower panel shows the change in confidence between the prior and posterior decisions.

Participants vary broadly in their reported confidence, both in terms of median confidence and the spread of reported values. This could be due to different subjective mappings between the salience of cues available to answer the question and the reported confidence^1^, but may also simply be due to different levels of expertise in the domains of the presented questions. Notably, the posterior decisions were generally made with higher degrees of confidence, as indicated by the lower panel of Figure 1. All participants had equal or higher median confidence in the posterior decisions.

Figure 1: Reported confidence levels of participants. Upper panel: Confidence reported before the conversation. Middle panel: Confidence reported after the conversation. Lower panel: Change in confidence from prior to posterior decision.


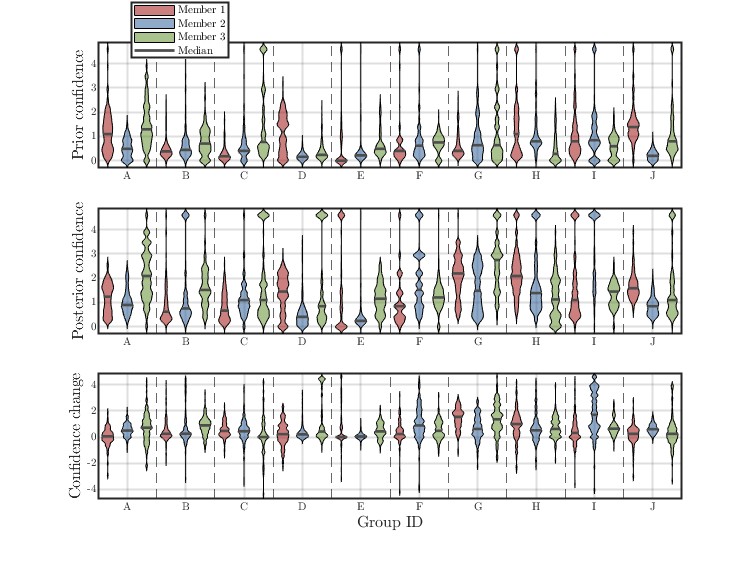


## Metacognitive calibration

When answering the questions, participants were asked to use the confidence scale such that their response would indicate their estimated probability of being correct. To investigate how successful participants were at doing this, linear regressions were performed to predict their binary score on each trial from their submitted confidence. For trials were participants submitted 50%, i.e. no preference for either option, the score was recorded as 0.5 (i.e. half of a correct answer). These regressions are shown in Figure 1, where each red line corresponds to one of the thirty participants. The solid black line shows the calibration across all participants, and the dashed black line indicates perfect calibration. The left and right panels show the pre- and post-conversation responses, respectively.

On average, participants tended to be fairly well-calibrated, such that their confidence ratings corresponded well with their scores, but there were substantial individual differences between participants. Some participants used only a small part of the scale and reported very low confidence ratings even when they were very likely to be correct, while others submitted maximal confidence ratings even when they were correct in as few as 65% of trials. This might suggest that at least some of the difference in the ranges of confidence ratings (see “Summary of confidence ratings by individuals in the supplementary materials) are due to differences in scale use and not just differences in domain knowledge.

There appears to be a tendency towards underconfidence at high levels of performance and overconfidence at low levels of performance. It is not uncommon to find biases in subjective confidence ratings, and many possible reasons for such effects have been proposed^2–4^.

Figure 1: Metacognitive calibration of prior (left panel) and posterior decisions (right panel). Red lines show individual participants, and the solid black line shows the average across participants. The dashed line indicates perfect calibration. On average, participants were fairly well-calibrated, but there are large variations between participants.


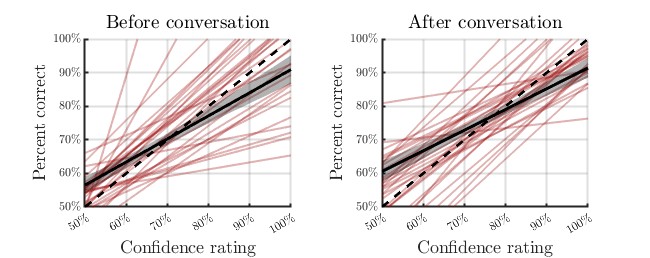


## Model evaluation and comparison

Each participant's model weights were estimated for each condition separately, using the estimator defined in Equation (3). In the main model, weights were estimated separately in each condition and for each member of each group, using a total of 84 trials (3 topics times 28 questions). To estimate the overall model fit, the model prediction error was evaluated using a ten-fold cross-validation of the root mean squared error (RMSE) of the predicted posterior confidence, $C^{p}$. The performance of the main model is shown in Figure 5 as the *Condition* model. In addition to the main model, the prediction error of three comparison models is also shown. The first comparison model, the *Group* model, predicted only one weight vector for each group, and was thus agnostic of both the condition and of individual group members’ identities. The second, the *Member* model, predicted individual weights for each group member, but was agnostic of the condition. The third model, the *List* model, further stratified the main model by also estimating separate weights for each of three topics. The models are ordered along the abscissa in Figure 1 such that the stratification level increases when moving to the right. Additionally, two baseline levels are shown as vertical lines. The first is the expected performance from random guessing of the posterior confidence (solid line). Random guesses were simulated as uniformly distributed values of $c^{p}$, which were then transformed into the log-odds domain for RMSE calculation. The second baseline model is the performance of a model fitted across all groups simultaneously using only a single weight vector (dashed line). This resulted in a weight of $[0.48 0.49 0.49]$, essentially a uniform weight with a constant scaling of the confidence magnitude. This baseline model is analogous to the one used Meyen et al. (2021)^5^, which also estimated a single set of parameters for a population of many distinct groups. The magnitude of the weight vector estimated here is very similar to the weight parameter found in that study, although it should be emphasized that the present study does not include the $\beta$-parameter from the original CWMV model.


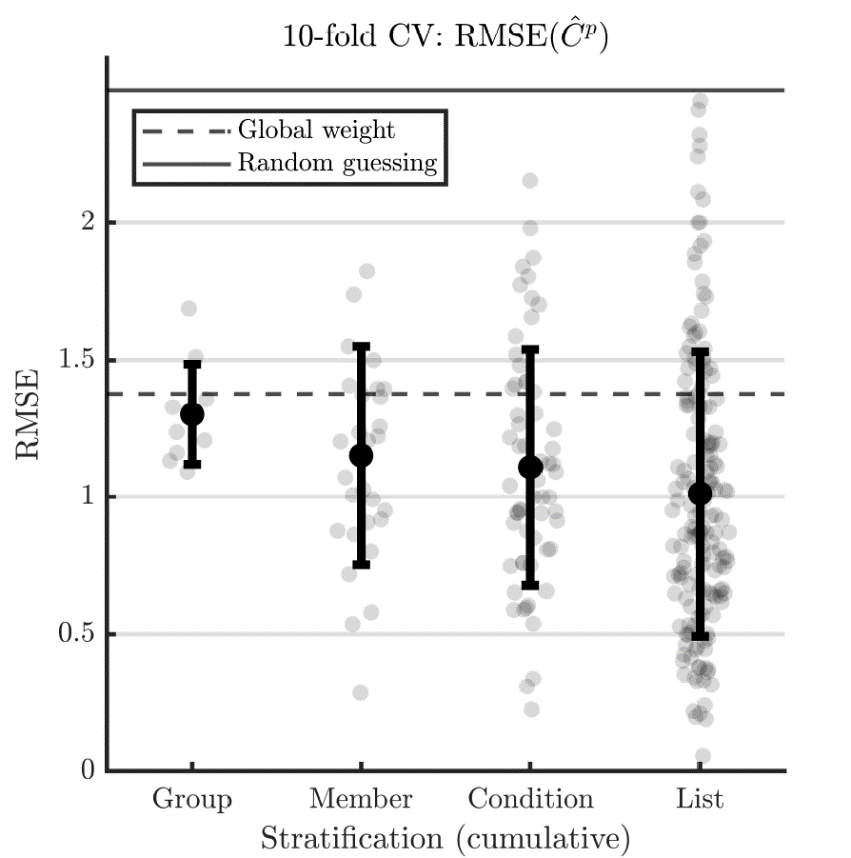


Figure 1: Out-of-sample prediction error (RMSE) of posterior confidence ratings $C^{p}$ using ten-fold cross-validation. Reported errors are the averages across the ten folds. Four model stratification levels are shown. Group: One weight vector per group, ten weight vectors in total. Member: One weight vector per group member, 30 in total. Condition: One weight vector per group member per condition, 60 in total. List: One weight per list (i.e., per conversation round), for a total of 180 weights.

All models performed much better than would be expected from random guessing. The *Member* and *Condition* models also generally outperform the single-weight baseline, indicating that subject-specific weights are beneficial for prediction. It is notable that the RMSE spread is large in these two models, suggesting substantial variability in how well the decision model fits participants’ actual behavior. The difference between the *Member* and *Condition* models is very small, indicating that condition-specific weights do not aid prediction. This does not preclude the possibility that individual participants' weights differ between conditions, but any such differences seem to be too small to benefit the prediction of posterior confidences at the population level. The *List* model performed slightly better than the *Member* and *Condition* models, suggesting that weights may also differ between the lists of each condition. However, the spread for this model is very large, stemming from the fact that only 25 or 26 trials are used to estimate each set of weights in each fold. Overall, this comparison shows that the *Condition* model performs on par or slightly better than the CWMV model in terms of prediction.

## Data availability statement

The confidence and decision data gathered from participants in this study is publicly available from DTU Data at DOI: 10.11583/DTU.25163816.

## References

1. Bang, D. *et al.* Does interaction matter? Testing whether a confidence heuristic can replace interaction in collective decision-making. *Conscious. Cogn.* **26**, 13–23 (2014).

2. Gigerenzer, G., Hoffrage, U. & Kleinbfilting, H. Probabilistic Mental Models: A Brunswikian Theory of Confidence.

3. Olsson, H. Measuring overconfidence: Methodological problems and statistical artifacts. *J. Bus. Res.* **67**, 1766–1770 (2014).

4. Juslin, P., Winman, A. & Olsson, H. Naive empiricism and dogmatism in confidence research: A critical examination of the hard–easy effect. *Psychol. Rev.* **107**, 384–396 (2000).

5. Meyen, S., Sigg, D. M. B., Luxburg, U. von & Franz, V. H. Group decisions based on confidence weighted majority voting. *Cogn. Res. Princ. Implic.* **6**, 18 (2021).
